# Discordant Prenatal Cell-Free DNA Screening vs. Diagnostic Results of Sex Chromosome Aneuploidies: Implications for Newborn Screening and Genetic Counseling

**DOI:** 10.3390/ijns10030048

**Published:** 2024-07-10

**Authors:** Susan Howell, Shanlee M. Davis, Billie Carstens, Mary Haag, Judith L. Ross, Nicole R. Tartaglia

**Affiliations:** 1 Department of Pediatrics, University of Colorado School of Medicine, Aurora, CO 80045, USA; shanlee.davis@cuanschutz.edu (S.M.D.); nicole.tartaglia@cuanschutz.edu (N.R.T.); 2 eXtraOrdinarY Kids Clinic and Research Program, Children’s Hospital Colorado, Aurora, CO 80045, USA; 3 Department of Pathology, University of Colorado School of Medicine, Aurora, CO 80045, USA; billie.carstens@cuanschutz.edu (B.C.); mary.haag@cuanschutz.edu (M.H.); 4 Department of Pediatrics, Nemours Children’s Hospital-DE and Thomas Jefferson University, Wilmington, DE 19803, USA; judith.ross@nemours.org

**Keywords:** newborn, sex chromosome aneuploidy, prenatal screening, cfDNA, discordance, genetic counseling, genetic testing, prenatal diagnosis, trisomy, tetrasomy

## Abstract

Sex chromosome aneuploidies (SCAs) collectively occur in 1 in 500 livebirths, and diagnoses in the neonatal period are increasing with advancements in prenatal and early genetic testing. Inevitably, SCA will be identified on either routine prenatal or newborn screening in the near future. Tetrasomy SCAs are rare, manifesting more significant phenotypes compared to trisomies. Prenatal cell-free DNA (cfDNA) screening has been demonstrated to have relatively poor positive predictive values (PPV) in SCAs, directing genetic counseling discussions towards false-positive likelihood rather than thoroughly addressing all possible outcomes and phenotypes, respectively. The eXtraordinarY Babies study is a natural history study of children prenatally identified with SCAs, and it developed a longitudinal data resource and common data elements with the Newborn Screening Translational Research Network (NBSTRN). A review of cfDNA and diagnostic reports from participants identified a higher than anticipated rate of discordance. The aims of this project are to (1) compare our findings to outcomes from a regional clinical cytogenetic laboratory and (2) describe discordant outcomes from both samples. Twenty-one (10%), and seven (8.3%) cases were found to be discordant between cfDNA (result or indication reported to lab) and diagnosis for the Babies Study and regional laboratory, respectively. Discordant results represented six distinct discordance categories when comparing cfDNA to diagnostic results, with the largest groups being Trisomy cfDNA vs. Tetrasomy diagnosis (66.7% of discordance in eXtraordinarY Babies study) and Mosaicism (57.1% in regional laboratory). Traditional genetic counseling for SCA-related cfDNA results is inadequate given a high degree of discordance that jeopardizes the accuracy of the information discussed and informed decision making following prenatal genetic counseling.

## 1. Introduction

Sex chromosome aneuploidies (SCAs), including Klinefelter syndrome/47,XXY, Trisomy X/47,XXX, and 47,XYY syndrome, are the most prevalent supernumerary chromosomal conditions, collectively occurring in approximately 1/500 livebirths. Clinical phenotypes are highly variable in these conditions, often with mild dysmorphic features or neurodevelopmental involvement, historically resulting in only 10–25% lifetime ascertainment. The majority of SCA diagnoses occur in the postnatal period during clinical evaluations for neurodevelopmental, medical, or infertility concerns [1]. However, given an increased risk for the early presentation of neurodevelopmental features in individuals with SCA, which were consistently described in prospective newborn studies of the 1970s, there is increasing discussion about whether universal newborn screening may allow for the initiation of early interventions and improve long-term outcomes [2,3,4]. While there have been no SCA neonatal screening studies since the 1970s, we now have an opportunity to learn from the many individuals who are being prenatally identified. The commercialization of prenatal cell-free DNA screening (cfDNA), also referred to as noninvasive prenatal screening, and its adoption into standardized U.S. obstetric care in 2016 has led to an immense increase in prenatally identified SCAs; however, confirmatory diagnostic testing is most frequently deferred to the postnatal period [5,6].

The growing utilization of cfDNA and increasing prenatal ascertainment of SCAs inspired the initiation of the eXtraordinarY Babies Study, a prospective natural history study of infants prenatally identified and subsequently diagnosed with SCA, designed to examine trajectories of neurodevelopment and physical health from birth throughout childhood as well as psychosocial factors, including quality of life and parental experiences [7]. Given the high phenotypic variability in SCAs, a primary aim of this study is to evaluate predictors of outcomes that establish rigorous evidence of effective interventions, thus impacting long-term outcomes and informing the future foundation for newborn screening. Funded by the National Institute of Child Health and Human Development (NICHD) (ClinicalTrials.gov (accessed on 5 April 2024), NCT03396562), the eXtraordinarY Babies Study enrolls infants under one year of age with a prenatally identified and subsequent prenatal/postnatal diagnosis of SCA. Longitudinal evaluations are conducted at two sites, including the University of Colorado/Children’s Hospital Colorado and Nemours-Children’s Hospital-DE. Longitudinal question and answer sets were developed with the Newborn Screening Translational Research Network (NBSTRN) [8] and expanded the neurodevelopmental common data elements (CDEs) in the Longitudinal Pediatric Data Resource (LPDR). While the eXtraordinarY Babies Study aims to prospectively describe and compare the natural history of SCA conditions, identify predictors of outcomes in SCA, and build a rich dataset linked to a biobank for future study, much has also been learned about the cfDNA results and experience, including discordance, prior to SCA diagnosis in study participants [7].

While cfDNA has historically demonstrated a high sensitivity, specificity and positive predictive value (PPV) in identifying autosomal aneuploidies, such as Trisomy 21/Down syndrome, the PPV for the detection of SCAs remains relatively poor, impacting prenatal genetic counseling [9,10,11,12]. Recent meta-analysis publications address and support this universal limitation of cfDNA in SCAs, with reported PPVs in the cfDNA detection of SCAs ranging between 15–32% in 45,X; 68–98% in 47,XXY; 58–62% in 47,XXX; and 71–100% in 47,XYY [13,14,15]. However, cfDNA for SCAs continues to be a strongly recommended prenatal screening option for patients, as endorsed by the American College of Medical Genetics and Genomics’ most recent clinical guidelines [16]. Therefore, genetic counseling for SCA-positive cfDNA results risks overemphasizing that cfDNA results cannot be considered diagnostic given PPVs, thereby limiting time and unbiased attention to address a thorough discussion of all possible outcomes and respective phenotypes, in order to facilitate patients making the most informed decisions [17]. The aims of this project are to compare our study findings to outcomes from a regional clinical cytogenetic laboratory, examining 21 of 209 and 8 of 84 cases of discordance, respectively, as well as to explore genetic counseling implications to further inform prenatal genetic counseling.

## 2. Materials and Methods

Participants from the eXtraordinarY Babies Study previously provided written consent (approval for human subjects research by Colorado COMIRB#17-0118 and Nemours Office of Human Subjects Protection #1151006; NIH/NICHD# R01HD42974; ClinicalTrials.gov# NCT03396562). Inclusion criteria for the eXtraordinarY Babies Study: Diagnosis of SCA before or at birth with enrollment prior to 13 months of age. Exclusion criteria: birth < 34 weeks, mosaicism > 20%, additional genetic/metabolic disorder with neurodevelopmental or endocrine involvement, congenital malformation (not previously described with SCA), or neonatal complications such as hypoxic-ischemic brain injury or neonatal meningitis. This analysis includes participants of the eXtraordinarY Babies Study for whom diagnostic cytogenetic testing (prenatal and/or postnatal) followed cfDNA and for whom both lab reports were available for review to determine discordance.

Data were abstracted from the eXtraordinarY Babies Study, including family history (maternal date of birth to calculate age at delivery, maternal height and pre-pregnancy weight to calculate BMI), diagnostic history by clinical interview (cfDNA, prenatal history, diagnostic timing) and review of genetic lab reports (including fetal fraction on cfDNA reports, when provided) conducted by a board-certified genetic counselor (SH). Data from the regional clinical cytogenetics laboratory of specific cases with an indication of cfDNA+ for SCA were provided for the same time-period as the eXtraordinarY Babies Study (2017–2023) and de-identified, including the biospecimen type analyzed and final karyotype result.

A discordant result was defined if the cfDNA result was different and abnormal from the diagnostic cytogenetic result. cfDNA results assigned broad categories, such as “atypical” or “inconclusive”, were considered true positives in cases of SCA diagnoses. Participants were excluded if either report could not be obtained for review. Descriptive statistics were utilized to describe the sample and summarize the data. 

## 3. Results

A total of 209 participants in the eXtraordinarY Babies study with a history of cfDNA and 84 results with an indication of cfDNA+ or atypical for SCA from the regional clinical cytogenetic laboratory were included in analyses. Both groups demonstrated the largest cohort of true positives to be 47,XXY (61.2% and 45.7%, respectively), followed by 47,XXX and 47,XYY (Table 1). The regional clinical cytogenetic laboratory identified 36.9% false positives (typical karyotype following abnormal cfDNA), of which the most common cfDNA results were atypical for sex chromosomes (48.4%), increased risk for 47,XXX (22.6%) and increased risk for 47,XXY (16.1%). The eXtraordinarY Babies study participants did not include anyone with 46,XX or 46,XY cytogenetic results, as defined by inclusion criteria.

With regard to discordance, 10% (*n* = 21) of the eXtraordinarY Babies study participants were classified as discordant when compared to diagnostic cytogenetic results. The confirmed diagnoses of these 21 infants included XXYY (*n* = 11), XXY (*n* = 2), XXX (*n* = 2), XXXY (*n* = 2), XYY (*n* = 1), XXXX (*n* = 1) and mosaic (*n* = 2), as shown in Table 1. 

Discordance categories identified among the 21 results included (in rank order):

1. cfDNA SCA trisomy result with SCA tetrasomy diagnosed (*n* = 14)
2. cfDNA trisomy result with mosaic SCA including a 46,XX or 46,XY cell line diagnosed (*n* = 2)
3. cfDNA autosomal trisomy result with SCA trisomy diagnosed (*n* = 2)
4. cfDNA monosomy X result with trisomy X diagnosed (*n* = 2)
5. cfDNA SCA trisomy with different SCA trisomy diagnosed (*n* = 1)
6. cfDNA SCA trisomy result with X;Y chromosome translocation diagnosed (none)

Of the 84 outcomes following an cfDNA result at risk for SCA, 8.3% (*n* = 7) were classified as discordant when compared to the diagnostic cytogenetic results, with the majority of discordance attributed to mosaic cytogenetic findings (*n* = 4; 57.1%) followed by structural X;Y chromosome translocations (*n* = 2; 28.6%).

Discordance categories identified among the eight discordant results included (in rank order):

1. cfDNA trisomy result with mosaic SCA including a 46,XX or 46,XY cell line diagnosed (*n* = 4)
2. cfDNA SCA trisomy result with X;Y chromosome translocation diagnosed (*n* = 2)
3. cfDNA SCA trisomy result with SCA tetrasomy diagnosed (*n* = 1)
4. cfDNA autosomal trisomy result with SCA trisomy diagnosed (none)
5. cfDNA monosomy X result with trisomy X diagnosed (none)
6. cfDNA SCA trisomy with different SCA trisomy diagnosed (none)

**Table 1:** Prenatal cell-free DNA screening (cfDNA) + Sex Chromosome Aneuploidy (SCA) outcomes in eXtraordinarY Babies Study vs. Regional Clinical Cytogenetics Laboratory.

cfDNA vs. Cyto	eXtraordinarY Babies Study (Total = 209)	Regional Clinical Cytogenetic Lab (Total = 84)
(cfDNA result; Karyotype)	*n* (%)	*n* (%)
True Positive	188 (90.0)	46 (54.8)
Atypical/XXY	115 (61.2)	21 (45.7)
Atypical/XXX	41 (21.8)	15 (17.6)
Atypical/XYY	32 (17.0)	10 (11.8)
False Positive	n/a	31 (36.9)
Atypical; 46,XX	-	9 (29.0)
XXX; 46,XX	-	7 (22.6)
Atypical; 46,XY	-	6 (19.4)
XXY; 46,XY	-	5 (16.1)
Y by cfDNA; 46,XX	-	3 (9.7)
Monosomy X; 46,XX	-	1 (3.2)
Discordant Abnl cfDNA & Abnl Cyto	21 (10.0)	7 (8.3)
Trisomy; Tetrasomy	14 (66.7)	1 (14.3)
SCA Trisomy/Atypical; Mosaic	2 (9.5)	4 (57.1)
SCA Trisomy; Translocation (X;Y)	-	2 (28.6)
Monosomy; trisomy	2 (9.5)	-
Autosomal Trisomy; SCA Trisomy	2 (9.5)	-
SCA Trisomy; Other SCA Trisomy	1 (4.8)	-

The largest (66.7%) discordant category for the eXtraordinarY Babies study was cfDNA-resulting SCA trisomy and cytogenetics-confirmed SCA tetrasomy, whereas the regional clinical cytogenetics laboratory identified only a single case (14.3%) in this discordant category. The largest (57.1%) discordant category for the regional clinical cytogenetics laboratory was cfDNA-resulting SCA trisomy/atypical and cytogenetics-confirmed fetal mosaicism, whereas the eXtraordinarY Babies study only identified two infants (9.5%) in this discordant category; however, mosaicism > 20% was exclusionary.

Fourteen discordant cases were confirmed to have SCA tetrasomy in the eXtraordinarY Babies study, as shown in Table 2. The majority of these were confirmed to be 48,XXYY (*n* = 12, 85.7%) and declined prenatal diagnostic procedures with postnatal cytogenetic testing (*n* = 13, 92.9%). Given the clinical implications of tetrasomy vs. trisomy, fetal fraction, maternal pre-pregnancy BMI and maternal age at birth were included for analysis, as these factors have been associated with prenatal cfDNA test validation and reliability. The median fetal fraction was 6.55% (range 4–19; common minimum cut-off 4%, decreases with higher maternal BMI and age [18,19]), and maternal age ranges at delivery were mostly 30–34 years (35.7%) and 35–39 years (21.4%). The mean maternal pre-pregnancy BMI was 27.6 (IQR 19.3–30.2). Prenatal information for the tetrasomy discordant case identified by the regional clinical cytogenetic laboratory was not available.

## 4. Discussion

The eXtraordinarY Babies study is one of the largest to date presenting a series of 21 infants (10.0% of our 209 infant sample) with a history of prenatal cfDNA results discordant from their final SCA diagnosis. When compared with cytogenetic outcomes of 84 cases referred to a regional clinical cytogenetic laboratory for testing over the same time period due to indications of cfDNA+ for SCA, 54.8% were confirmed true positives, of which 8.3% were identified as discordant, yet for different reasons. Our findings unanimously emphasize the importance of comprehensive prenatal genetic counseling for cfDNA+ results addressing all possible outcomes, including discordance, to appropriately support a patient’s ability to make a true informed decision.

Discordance between cfDNA results and fetal karyotype has been well established to be attributed to various factors, such as confined placental mosaicism, maternal copy number variations (CNVs), maternal X chromosome aneuploidy and/or mosaicism, maternal malignancy, vanishing twin, and technical, bioinformatics, or human errors [20]. In a recent publication by Tang et al., data collected over 5 years identified 71 of 177 (40%) women with false-positive cfDNA results for fetal sex chromosome aneuploidies, of which 23 (32%) false-positives were subsequently determined to be attributed to maternal factors, including maternal mosaic monosomy X as well as maternal mosaic and non-mosaic 47,XXX [21]. Maternal aneuploidy (mosaic and non-mosaic) incidental findings not only have medical and psychological implications for the mother given her new diagnosis, but also inform the mother of potential future reproductive issues and serve to guide appropriate prenatal diagnosis strategies for future pregnancies. Collectively, these aspects highlight the importance of comprehensive prenatal genetic counseling for SCA+ cfDNA results, unbiased by a discussion of PPV, as potential diagnostic outcomes warrant informed decisions with significant implications not only for the fetus, but also for the mother.

Categorically, the largest discordance in our eXtraordinarY Babies study sample, which was also an observed outcome from the regional clinical cytogenetics lab, was cfDNA+ results for SCA trisomy with SCA tetrasomy diagnosed (*n* = 14, 66.7% of all discordant cases), in which prenatal counseling risks inaccuracy if solely based upon the cfDNA+ resulted condition. A risk that is endorsed when cfDNA reports label the result specific to a trisomy (“XXY”) elaborates on symptoms associated with the trisomy condition (“What is XXY”) and fails to highlight the possibility of a discordant diagnostic outcome. Tetrasomy-discordant outcomes present the most significant risk for inaccurate prenatal counseling, if counseling is based solely on the trisomy SCA+ per cfDNA report. Tetrasomies are well-established as presenting with more severe medical, developmental and psychological phenotypes when compared to trisomies, including, but not limited to, congenital malformations [22,23]. A similar risk also applies for diagnosed cfDNA+ autosomal trisomy and SCA trisomy, as autosomal trisomies also present with more significant medical and neurodevelopmental phenotypes compared to SCA trisomies. However, cfDNA+ for an autosomal trisomy or monosomy X result risks subsequent targeted ultrasounds for associated sonographic markers, which could be falsely reassuring [24,25,26].

The eXtraordinarY Babies study findings also highlight discordant results in which cytogenetic testing was deferred to the postnatal period, further underscoring the importance of discussing possible discordant outcomes during genetic counseling on cfDNA+ results. Prior studies have investigated patient decision-making following cfDNA+ SCA results and reported that more than half of patients will decline prenatal diagnostic procedures and elect to pursue cytogenetic testing postnatally [5,27]. While PPVs are relatively poor in cfDNA for SCA, genetic counseling overemphasizing a PPV, and most likely a false positive, risks biased counseling and shortcomings in honoring reproductive autonomy with fully informed decision-making.

Globally, professional societies do not all universally recommend cfDNA including SCAs. Some European societies that do not endorse cfDNA for SCAs emphasize perspectives of raised ethical concerns, including a decreased clinical utility in the context of suggested mild phenotypic outcomes and relatively poor PPVs as well as limited equity of access from a public health perspective [28,29,30]. In a recent publication by Johnston et al. evaluating ethical principles relevant to cfDNA in SCA, they concluded that cfDNA for SCA should be universally offered to patients based on the overarching ethical principle of reproductive autonomy and emphasized that the onus of addressing the limitations of cfDNA in SCAs be placed on the providers during more comprehensive genetic counseling [30]. One possible solution to address this global debate would be pivoting from cfDNA to newborn screening for SCAs, supported by predictors of outcomes evaluated in the eXtraordinarY Babies Study and other studies evaluating early neurodevelopmental phenotypes that have resulted in evidence of risk factors and effective interventions impacting long-term outcomes for patients with SCA [31,32,33,34,35].

Currently, conditions that the US Department of Health and Human Services include as part of the Recommended Uniform Screening Panel (RUSP) require evidence of a net benefit of screening and the availability of effective treatments, as well as the availability of a cost-effective test to screen for the disorder [36]. While this study supports the net benefits of universal newborn screening vs. prenatal cfDNA screening, given risks of potential harm as well as inadequate counsel due to relatively poor PPV for prenatal cfDNA and possible discordant results, the reality is that prenatal cfDNA screening has already been adopted in clinical practice and that the transition to genetic-based NBS tests that will incidentally identify SCA conditions is not far behind. Acknowledging this future landscape, whether or not current evidence to support treatment efficacy and/or cost-effectiveness is available, warrants attention. The more critical question is to establish rigorous evidence of effective early intervention for persons with SCA that will impact long-term outcomes [37,38,39,40]. This argument is consistent with many proponents of an early diagnosis of neurogenetic developmental disorders in order to direct an early initiation of developmental therapies, prevent the diagnostic odyssey, and improve outcomes, even when not curative [41]. Given the context of misinformed counsel with discordant cfDNA results that could undermine autonomous decision-making and likely cause harm from confusion and distress, combined with modifiable predictors of outcome and effective postnatal treatment interventions improving long-term outcomes, incorporating SCAs as part of the RUSP should be strongly considered [42].

Our findings of various categories of discordance by cfDNA both in the eXtraordinarY Babies study and regional clinical cytogenetic laboratory, combined with the well-established parental decision preference to defer to postnatal confirmation, suggest that prenatal genetic counseling on cfDNA+ results may be insufficient. While limitations in our results reflect possible ascertainment bias of our study cohort, further research directions aim to investigate the decisional impacts of quoted PPV, cfDNA report terminology, post-cfDNA counseling, and sonographic results, when applicable.

## Figures and Tables

**Table 2:** cfDNA+ SCA trisomy with tetrasomy diagnostic outcomes in eXtraordinarY Babies Study.

cfDNA Result	Cytogenetic Result	Fetal Fraction (%)	Maternal Pre-Pregnancy BMI (kg/cm^2^)	Maternal Age Range at Birth (yrs)
XXY	48,XXYY	7.0	26.5	30–34
XXY	48,XXYY	NR	22.4	30–34
XXY	48,XXYY	4.0	36.6	35–39
XYY	48,XXYY	6.0	25.6	20–24
XXY	48,XXYY	6.1	30.2	30–34
XXY	48,XXYY	NR	22.1	45–49
XXY	48,XXYY	13.0	28.3	30–34
XXY	48,XXYY	11.0	20.4	30–34
XXY	48,XXYY	4.8	27.2	20–24
XXY	48,XXYY	7.2	NR	35–39
XXY	48,XXXY	5.0	39	25–29
XYY	48,XXYY	7.1	24	20–24
XXY	48,XXYY	19.0	37.5	35–39
XXY	48,XXXY	6.0	19.3	15–19
		Median: 6.5 Range 4–19	Mean 27.6 IQR: 19.3, 30.2	Mode: 30–34

NR = Not Reported, cfDNA = Prenatal Cell-Free DNA Screening, SCA = Sex Chromosome Aneuploidy, BMI = Body Mass Index.

## Data Availability

Deidentified data that support the findings of this study are available from the corresponding author upon reasonable request.

## References

[1] Bojesen A., Juul S., Gravholt C.H. (2003). Prenatal and postnatal prevalence of Klinefelter syndrome: A national registry study. J. Clin. Endocrinol. Metab..

[2] Tennes K., Puck M., Orfanakis D., Robinson A. (1977). The early childhood development of 17 boys with sex chromosome anomalies: A prospective study. Pediatrics.

[3] Leonard M.F., Schowalter J.E., Landy G., Ruddle F.H., Lubs H.A. (1979). Chromosomal abnormalities in the New Haven newborn study: A prospective study of development of children with sex chromosome anomalies. Birth Defects Orig. Artic. Ser..

[4] Leonard M.F., Sparrow S. (1986). Prospective study of development of children with sex chromosome anomalies: New Haven Study IV. Adolescence. Birth Defects Orig. Artic. Ser..

[5] Howell S., Davis S.M., Thompson T., Brown M., Tanda T., Kowal K., Alston A., Ross J., Tartaglia N.R. (2023). Noninvasive prenatal screening (NIPS) results for participants of the eXtraordinarY babies study: Screening, counseling, diagnosis, and discordance. J. Genet. Couns..

[6] Gregg A.R., Skotko B.G., Benkendorf J.L., Monaghan K.G., Bajaj K., Best R.G., Klugman S., Watson M.S. (2016). Noninvasive prenatal screening for fetal aneuploidy, 2016 update: A position statement of the American College of Medical Genetics and Genomics. Genet Med..

[7] Tartaglia N., Howell S., Davis S., Kowal K., Tanda T., Brown M., Boada C., Alston A., Crawford L., Thompson T. (2020). Early neurodevelopmental and medical profile in children with sex chromosome trisomies: Background for the prospective eXtraordinarY babies study to identify early risk factors and targets for intervention. Am. J. Med. Genet. C Semin. Med. Genet..

[8] Chan K., Hu Z., Bush L.W., Cope H., Holm I.A., Kingsmore S.F., Wilhelm K., Scharfe C., Brower A. (2023). NBSTRN Tools to Advance Newborn Screening Research and Support Newborn Screening Stakeholders. Int. J. Neonatal Screen..

[9] Shi Y., Li X., Ju D., Li Y., Zhang X., Zhang Y. (2021). Efficiency of Noninvasive Prenatal Testing for Sex Chromosome Aneuploidies. Gynecol. Obstet. Investig..

[10] Lu X., Wang C., Sun Y., Tang J., Tong K., Zhu J. (2021). Noninvasive prenatal testing for assessing foetal sex chromosome aneuploidy: A retrospective study of 45,773 cases. Mol. Cytogenet..

[11] Zheng Y., Wan S., Dang Y., Song T., Chen B., Zhang J. (2020). Clinical experience regarding the accuracy of NIPT in the detection of sex chromosome abnormality. J. Gene Med..

[12] Skotko B.G., Allyse M.A., Bajaj K., Best R.G., Klugman S., Leach M., Meredith S., Michie M., Stoll K., Gregg A.R. (2019). Adherence of cell-free DNA noninvasive prenatal screens to ACMG recommendations. Genet. Med..

[13] Bussolaro S., Raymond Y.C., Acreman M.L., Guido M., Da Silva Costa F., Rolnik D.L., Fantasia I. (2023). The accuracy of prenatal cell-free DNA screening for sex chromosome abnormalities: A systematic review and meta-analysis. Am. J. Obstet. Gynecol. MFM.

[14] Shear M.A., Swanson K., Garg R., Jelin A.C., Boscardin J., Norton M.E., Sparks T.N. (2023). A systematic review and meta-analysis of cell-free DNA testing for detection of fetal sex chromosome aneuploidy. Prenat. Diagn..

[15] Soukkhaphone B., Lindsay C., Langlois S., Little J., Rousseau F., Reinharz D. (2021). Non-invasive prenatal testing for the prenatal screening of sex chromosome aneuploidies: A systematic review and meta-analysis of diagnostic test accuracy studies. Mol. Genet. Genomic Med..

[16] Dungan J.S., Klugman S., Darilek S., Malinowski J., Akkari Y.M.N., Monaghan K.G., Erwin A., Best R.G., ACMG Board of Directors (2023). Noninvasive prenatal screening (NIPS) for fetal chromosome abnormalities in a general-risk population: An evidence-based clinical guideline of the American College of Medical Genetics and Genomics (ACMG). Genet. Med..

[17] Neufeld-Kaiser W.A., Cheng E.Y., Liu Y.J. (2015). Positive predictive value of non-invasive prenatal screening for fetal chromosome disorders using cell-free DNA in maternal serum: Independent clinical experience of a tertiary referral center. BMC Med..

[18] Fiorentino F., Bono S., Pizzuti F., Mariano M., Polverari A., Duca S., Sessa M., Baldi M., Diano L., Spinella F. (2016). The importance of determining the limit of detection of non-invasive prenatal testing methods. Prenat. Diagn..

[19] Deng C., Liu J., Liu S., Liu H., Bai T., Jing X., Xia T., Liu Y., Cheng J., Wei X. (2023). Maternal and fetal factors influencing fetal fraction: A retrospective analysis of 153,306 pregnant women undergoing noninvasive prenatal screening. Front. Pediatr..

[20] Hartwig T.S., Ambye L., Sorensen S., Jorgensen F.S. (2017). Discordant non-invasive prenatal testing (NIPT)—A systematic review. Prenat. Diagn..

[21] Tang X., Du Y., Chen M., Zhang Y., Wang Z., Zhang F., Tan J., Yin T., Wang L. (2024). Relationships among maternal monosomy X mosaicism, maternal trisomy, and discordant sex chromosome aneuploidies. Clin. Chim. Acta.

[22] Tartaglia N., Ayari N., Howell S., D’Epagnier C., Zeitler P. (2011). 48,XXYY, 48,XXXY and 49,XXXXY syndromes: Not just variants of Klinefelter syndrome. Acta Paediatr..

[23] Ricciardi G., Cammisa L., Bove R., Picchiotti G., Spaziani M., Isidori A.M., Aceti F., Giacchetti N., Romani M., Sogos C. (2022). Clinical, Cognitive and Neurodevelopmental Profile in Tetrasomies and Pentasomies: A Systematic Review. Children.

[24] Shi X.M., Fang Q., Chen B.J., Xie H.N., Xie Y.J., Chen J.H., Wu J.Z. (2013). Investigation of ultrasound markers in screening fetal trisomy 21. Zhonghua Fu Chan Ke Za Zhi.

[25] DeVore G.R. (2000). Trisomy 21: 91% detection rate using second-trimester ultrasound markers. Ultrasound Obstet. Gynecol..

[26] Swanson K., Bishop J.C., Al-Kouatly H.B., Makhamreh M., Felton T., Vora N.L., Sparks T.N., Jelin A.C. (2023). Prenatal phenotype of 47, XXY (Klinefelter syndrome). Prenat. Diagn..

[27] Ramdaney A., Hoskovec J., Harkenrider J., Soto E., Murphy L. (2018). Clinical experience with sex chromosome aneuploidies detected by noninvasive prenatal testing (NIPT): Accuracy and patient decision-making. Prenat. Diagn..

[28] Dondorp W., de Wert G., Bombard Y., Bianchi D.W., Bergmann C., Borry P., Chitty L.S., Fellmann F., Forzano F., Hall A. (2015). Non-invasive prenatal testing for aneuploidy and beyond: Challenges of responsible innovation in prenatal screening. Eur. J. Hum. Genet..

[29] Gadsboll K., Petersen O.B., Gatinois V., Strange H., Jacobsson B., Wapner R., Vermeesch J.R., Group N.I.-m.S., Vogel I. (2020). Current use of noninvasive prenatal testing in Europe, Australia and the USA: A graphical presentation. Acta Obstet. Gynecol. Scand..

[30] Johnston M., Warton C., Pertile M.D., Taylor-Sands M., Delatycki M.B., Hui L., Savulescu J., Mills C. (2023). Ethical issues associated with prenatal screening using non-invasive prenatal testing for sex chromosome aneuploidy. Prenat. Diagn..

[31] Bouw N., Swaab H., Tartaglia N., Wilson R.L., Van der Velde K., van Rijn S. (2023). Early symptoms of autism spectrum disorder (ASD) in 1-8 year old children with sex chromosome trisomies (XXX, XXY, XYY), and the predictive value of joint attention. Eur. Child. Adolesc. Psychiatry.

[32] Capelli E., Silibello G., Ajmone P.F., Altamore E., Lalatta F., Vizziello P.G., Costantino M.A., Zampini L. (2022). Language Development in Sex Chromosome Trisomies: Developmental Profiles at 2 and 4 Years of Age, and Predictive Measures. Dev. Neurorehabil.

[33] Urbanus E., Swaab H., Tartaglia N., Stumpel C., van Rijn S. (2023). Structural and pragmatic language in young children with sex chromosome trisomy (XXX, XXY, XYY): Predictive value for neurobehavioral problems one year later. Clin. Neuropsychol..

[34] Capelli E., Silibello G., Provera A., Dall’Ara F., Ajmone P.F., Monti F., Scionti N., Zanchi P., Costantino M.A., Vizziello P.G. (2023). Speech Sound Development in 18-Month-Old Children With Sex Chromosome Trisomies. Am. J. Speech Lang. Pathol..

[35] Martin F., Swaab H., Bierman M., van Rijn S. (2023). Effectiveness of social management training on executive functions in males with Klinefelter syndrome (47, XXY). Appl. Neuropsychol. Adult.

[36] Hinton C.F., Homer C.J., Thompson A.A., Williams A., Hassell K.L., Feuchtbaum L., Berry S.A., Comeau A.M., Therrell B.L., Brower A. (2016). A framework for assessing outcomes from newborn screening: On the road to measuring its promise. Mol. Genet. Metab..

[37] Godler D.E., Inaba Y., Schwartz C.E., Bui Q.M., Shi E.Z., Li X., Herlihy A.S., Skinner C., Hagerman R.J., Francis D. (2015). Detection of skewed X-chromosome inactivation in Fragile X syndrome and X chromosome aneuploidy using quantitative melt analysis. Expert. Rev. Mol. Med..

[38] Coffee B., Keith K., Albizua I., Malone T., Mowrey J., Sherman S.L., Warren S.T. (2009). Incidence of fragile X syndrome by newborn screening for methylated FMR1 DNA. Am. J. Hum. Genet..

[39] Campos-Acevedo L.D., Ibarra-Ramirez M., de Jesus Lugo-Trampe J., de Jesus Zamudio-Osuna M., Torres-Munoz I., Del Roble Velasco-Campos M., Rojas-Patlan L., Rodriguez-Sanchez I.P., Martinez-de-Villarreal L.E. (2016). Dosage of Sex Chromosomal Genes in Blood Deposited on Filter Paper for Neonatal Screening of Sex Chromosome Aneuploidy. Genet. Test. Mol. Biomark..

[40] Grati F.R., Bajaj K., Zanatta V., Malvestiti F., Malvestiti B., Marcato L., Grimi B., Maggi F., Simoni G., Gross S.J. (2017). Implications of fetoplacental mosaicism on cell-free DNA testing for sex chromosome aneuploidies. Prenat. Diagn..

[41] Chung W.K., Berg J.S., Botkin J.R., Brenner S.E., Brosco J.P., Brothers K.B., Currier R.J., Gaviglio A., Kowtoniuk W.E., Olson C. (2022). Newborn screening for neurodevelopmental diseases: Are we there yet?. Am. J. Med. Genet. C Semin. Med. Genet..

[42] Gies I., Tournaye H., De Schepper J. (2016). Attitudes of parents of Klinefelter boys and pediatricians towards neonatal screening and fertility preservation techniques in Klinefelter syndrome. Eur. J. Pediatr..

